# Effects of intra-annual precipitation patterns on grassland productivity moderated by the dominant species phenology

**DOI:** 10.3389/fpls.2023.1142786

**Published:** 2023-04-11

**Authors:** Ze Zhang, Zhihao Zhang, Yann Hautier, Hua Qing, Jie Yang, Tiejun Bao, Olivia L. Hajek, Alan K. Knapp

**Affiliations:** ^1^ Ministry of Education Key Laboratory of Ecology and Resource Use of the Mongolian Plateau, Inner Mongolia University, Hohhot, China; ^2^ Inner Mongolia Key Laboratory of Grassland Ecology, School of Ecology and Environment, Inner Mongolia University, Hohhot, China; ^3^ Ecology and Biodiversity Group, Department of Biology, Utrecht University, Utrecht, Netherlands; ^4^ Department of Biology and Graduate Degree Program in Ecology, Colorado State University, Fort Collins, CO, United States

**Keywords:** soil moisture, green-up, flowering, senescence, above-ground biomass, complementary effect

## Abstract

Phenology and productivity are important functional indicators of grassland ecosystems. However, our understanding of how intra-annual precipitation patterns affect plant phenology and productivity in grasslands is still limited. Here, we conducted a two-year precipitation manipulation experiment to explore the responses of plant phenology and productivity to intra-annual precipitation patterns at the community and dominant species levels in a temperate grassland. We found that increased early growing season precipitation enhanced the above-ground biomass of the dominant rhizome grass, *Leymus chinensis*, by advancing its flowering date, while increased late growing season precipitation increased the above-ground biomass of the dominant bunchgrass, *Stipa grandis*, by delaying senescence. The complementary effects in phenology and biomass of the dominant species, *L. chinensis* and *S. grandis*, maintained stable dynamics of the community above-ground biomass under intra-annual precipitation pattern variations. Our results highlight the critical role that intra-annual precipitation and soil moisture patterns play in the phenology of temperate grasslands. By understanding the response of phenology to intra-annual precipitation patterns, we can more accurately predict the productivity of temperate grasslands under future climate change.

## Introduction

Phenology is the timing of periodic biological events of plants, and it is an important indicator of ecosystem dynamics, determining to a large extent community productivity and providing insight into species distribution (Estiarte and Peñuelas, 2015; Zelikova et al., 2015; Li et al., 2016a; Zhou et al., 2016). As globally widespread ecosystems (Ren et al., 2019), grasslands provide critical ecosystem functions and services (Smith and Knapp, 2003); understanding how climate change may shift the phenology of these ecosystems will be critical for predicting future functions. Previous work has demonstrated that grassland phenology is both an important predictor of intra-season biomass production and highly sensitive to climatic variability (Badeck et al., 2004; Ganjurjav et al., 2020; Zhang et al., 2022a). Thus, with climate change, shifts in the timing of water availability and rising temperatures are likely to alter grassland phenology with significant implications for carbon cycling, forage production, and biodiversity (Richardson et al., 2013; Hajek and Knapp, 2022).

The shifts in plant phenology over the last several decades provide compelling evidence that grassland ecosystems are already responding to climate change (Parmesan and Yohe, 2003; Shen et al., 2011; Barichivich et al., 2013; Richardson et al., 2013; Li et al., 2016b). Many studies have documented earlier green-up dates for grassland plants in response to climate warming (Yuan et al., 2007; Piao et al., 2011; Cleland et al., 2012; Xia and Wan, 2013; Ge et al., 2015; Mo et al., 2017; Moore and Lauenroth, 2017). However, semi-arid grassland ecosystems are more likely to be affected by changes in water availability than temperature (Niu et al., 2008; Vico et al., 2015; Li et al., 2017), so small changes in precipitation patterns may have large impacts on species phenology and productivity in these communities (Heisler-White et al., 2008; Post et al., 2022). For example, Wang et al. (2019) found that changes in spring precipitation correlate well with variations in spring phenology based on an analysis of long-term field phenological and climate records from eight grassland sites in Inner Mongolia. Similarly, a study that used satellite normalized difference vegetation index (NDVI) data to analyze spring phenology changes in temperate grasslands over a 33-year period found that the main driver of shifts in spring phenology was a significant increase in preseason precipitation (Fu et al., 2021). Further, recent studies have shown that changes in autumn phenology also contribute significantly to the extension of the growing season and accumulation of biomass with increased precipitation, modulating the dynamics of carbon cycling in semi-arid grasslands (Peñuelas et al., 2004; Piao et al., 2008; Garonna et al., 2014). Overall, there is mounting evidence that changes in precipitation alter the growing season length by either advancing the start, delaying the end of the growing season, or both.

However, it is still unclear how intra-annual precipitation variations affect plant phenology and productivity in grasslands, although understanding the seasonal precipitation sensitivity of the various plant functional groups may help to predict ecosystem responses to these changes (Bai et al., 2004; Hallett et al., 2019; Shaw et al., 2022). For example, Huenneke et al. (2002) reported that grass-dominated systems mainly use summer precipitation for flowering, while shrub-dominated systems often rely on spring precipitation for green-up. Other studies have found that decreased early growing season precipitation delays the green-up of grasses, whereas decreased late growing season precipitation delays the flowering dates of forbs (Zhang et al., 2020). Such variations in species phenology responses to climate change may eventually alter the total biomass at the end of the season due to differences in the seasonal growth patterns of different functional groups (Dudney et al., 2017; Zhang et al., 2022b).

Dominant species’ responses to seasonal precipitation shifts are particularly relevant, because these species make the greatest contribution to the total biomass of grassland ecosystems, and their phenological characteristics are closely linked to ecosystem functions, meaning that how dominant species respond to climate change will largely drive future grassland productivity (Grime, 1998; Smith and Knapp, 2003). Further, understanding how the phenology of dominant species from different functional groups responds to variations in intra-annual precipitation patterns may reveal potential mechanisms for grassland community productivity stability (Bai et al., 2004; Hillebrand et al., 2008; Gonzalez and Loreau, 2009; Hautier et al., 2015). Overall, research addressing how climate change will affect changes in phenology and the productivity of dominant species, and subsequently the community, is necessary to inform future management options for grassland ecosystems (Prevéy and Seastedt, 2014; Wang et al., 2019).

Here, we performed an experiment manipulating the intra-annual precipitation patterns from April to September during the growing seasons of 2020 and 2021 in a temperate grassland in north China. Temperate grasslands are an ideal ecosystem to evaluate the effects of intra-annual precipitation variation on dominant species/community phenology and productivity, because species turnover and composition patterns are sensitive to intra-annual precipitation fluctuations (Chen et al., 2005; Liu et al., 2022). The specific objectives of the study were to reveal: 1) how intra-annual precipitation patterns affect plant phenology at both the dominant species and community level, and 2) how these responses drive dominant species and community productivity. We hypothesize that the effects of intra-annual precipitation patterns on plant phenology and productivity depend on soil moisture, and the response of phenology and productivity to intra-annual precipitation patterns will be different among dominant species.

## Materials and methods

### Study site

The experiment was conducted in a temperate grassland (44°10’ N, 116°28′ E, 1101 m asl; **Supplementary Figure S1**) located in the Xilingol region of Inner Mongolia, China. The mean annual temperature is 0.77 °C, ranging from -21.2 °C in January to 19.4 °C in July. The mean annual precipitation is 300.2 mm, with 80% falling in the growing season (from April to September, Zhang et al., 2022b). The soil type is chestnut soil, according to the Chinese classification, with an average bulk density of 0-20 cm, specifically 1.3 g cm^−3^, and a pH of 7.7 (Yuan et al., 2005). Our experimental site, a 300 m × 300 m natural grassland, was fenced to exclude grazing in 2016. Prior to the initiation of our experiment, we harvested the community above-ground biomass within 50 individual 1 m × 1 m quadrats along two diagonal lines in early September 2019 (the period of peak biomass) to identify the dominant species. Dominant species were defined as species whose relative above-ground biomass accounted for > 5% of the total above-ground biomass of the community (Ma et al., 2017). Two dominant species were identified—one perennial rhizome grass, *Leymus chinensis* (41.2 ± 5.3%), and one perennial bunchgrass, *Stipa grandis* (35.7 ± 4.8%). The common and rare species within this experimental site and their relative abundance are presented in **Supplementary Table S1**.

### Experimental design

This experiment was conducted from early April to late September during the growing seasons of 2020 and 2021. It used a domed automatic shelter (for specification details, see Wan et al., 2022) to provide shelter and quantitative irrigation. It included three replicates of each of the following five treatments: control (CK, multi-year average precipitation for the growing season), decreased 50% precipitation for the entire growing season (DP), increased 50% precipitation for the entire growing season (IP), increased early growing season (from April to June) precipitation by 50% (IEP), and increased late growing season (from July to September) precipitation by 50% (ILP). All treatments were performed under a single domed automatic canopy.

The decision to exclude or increase 50% precipitation was made based on historical precipitation data over a period of 30 years (1981–2011). During that period, the highest early growing season precipitation occurred in 1992, which was 48.08% above the average precipitation, and the highest late growing season precipitation occurred in 1990, which was 47.90% above the 30-year average precipitation. Similarly, we analyzed the historical precipitation data to determine the average number of precipitation days, the amount of precipitation, and the frequency of daily precipitation occurring in each month of the growing season in order to set the dates and amount of precipitation for each irrigation (see **Supplementary Table S2**).

We attempted to only add water at times just before sunset to limit evaporation. At the end of the growing season, all the plots were unsheltered and received ambient rain and snow until the start of the next growing season. There were fifteen 4 m × 3 m plots randomly located with a spacing of 1.5 m between them. We inserted aluminum flashing to a 1 m depth around each plot to prevent lateral movement of the soil moisture.

### Soil moisture and soil temperature measurement

We recorded the soil moisture and soil temperature in the top 20 cm (the primary root zone; Jackson et al., 1996) automatically every 30 min during the experiment using an CR1000X Data Collection System (Campbell Scientific, Inc., Utah, USA). We averaged the soil moisture and soil temperature for each day and then used those values to calculate the daily averages for each treatment.

### Community biomass measurement

In early September each year, a 1 m^2^ quadrat was randomly located within each plot, and all plants above the soil surface were collected, sorted, and oven-dried to a constant weight. They were then weighed to the nearest 0.01 g, and the sum of the dry weights of all the species was the community above-ground biomass. The location of the quadrat was marked during each survey to avoid establishing a quadrat in the same place the following year.

### Phenology measurements

We selected the two dominant species, grass *L. chinensis* and *S. grandis*, which summed up to 76.8 ± 11.2% of the total community above-ground relative dry biomass of all the experimental plots, to measure phenological change. In each plot, five individuals of these two dominant species were randomly labeled and monitored for phenology at 7-day intervals from early April to late September in 2020 and 2021. We defined the date of leaf (or flower) emergence as green-up (or flowering) and the date of leaf autumn coloring as senescence. As 50% of the observed plants reached a phenological event, the date of each phenological event was recorded (Li et al., 2016a).

### Statistical analysis

Repeated-measures analysis of variance (RMANOVA) was used to examine the separate and interactive effects of year, growth stage, and intra-annual precipitation patterns on soil moisture and soil temperature. One-way ANOVAs were used to explore the differences in green-up, flowering, senescence, and above-ground biomass among intra-annual precipitation patterns, both at the species and community level. RMANOVA was employed to evaluate the separate and interactive effects of year, species, and intra-annual precipitation patterns on community green-up, flowering, senescence, and above-ground biomass. In this study, the weighted average of the relative above-ground biomass of the two monitored dominant species was used to represent the community-level phenology. All analyses were performed using the SPSS 19.0 software package.

Structural equation models (SEM) were used to explore the effects of soil moisture and soil temperature on above-ground biomass through shifts in plant phenology, including plant green-up, flowering, and senescence, at both the species and community level. An *a priori* model was developed based on the potential relationships between plant phenology and above-ground biomass (**Figure S2**). The initial model was simplified based on the regression weight estimates, and the final model contained only paths that were statistically significant. The model had a good accuracy and fit when chi-squared test *X ^2^* ≥ 0, P > 0.05, the root-mean-square errors of approximation (RMSEA) ≤ 0.08, and with the lowest Akaike information criterion (AIC) value. SEM was performed using the AMOS 22.0 software package.

## Results

### Changes in soil moisture and temperature

**Figure 1:**
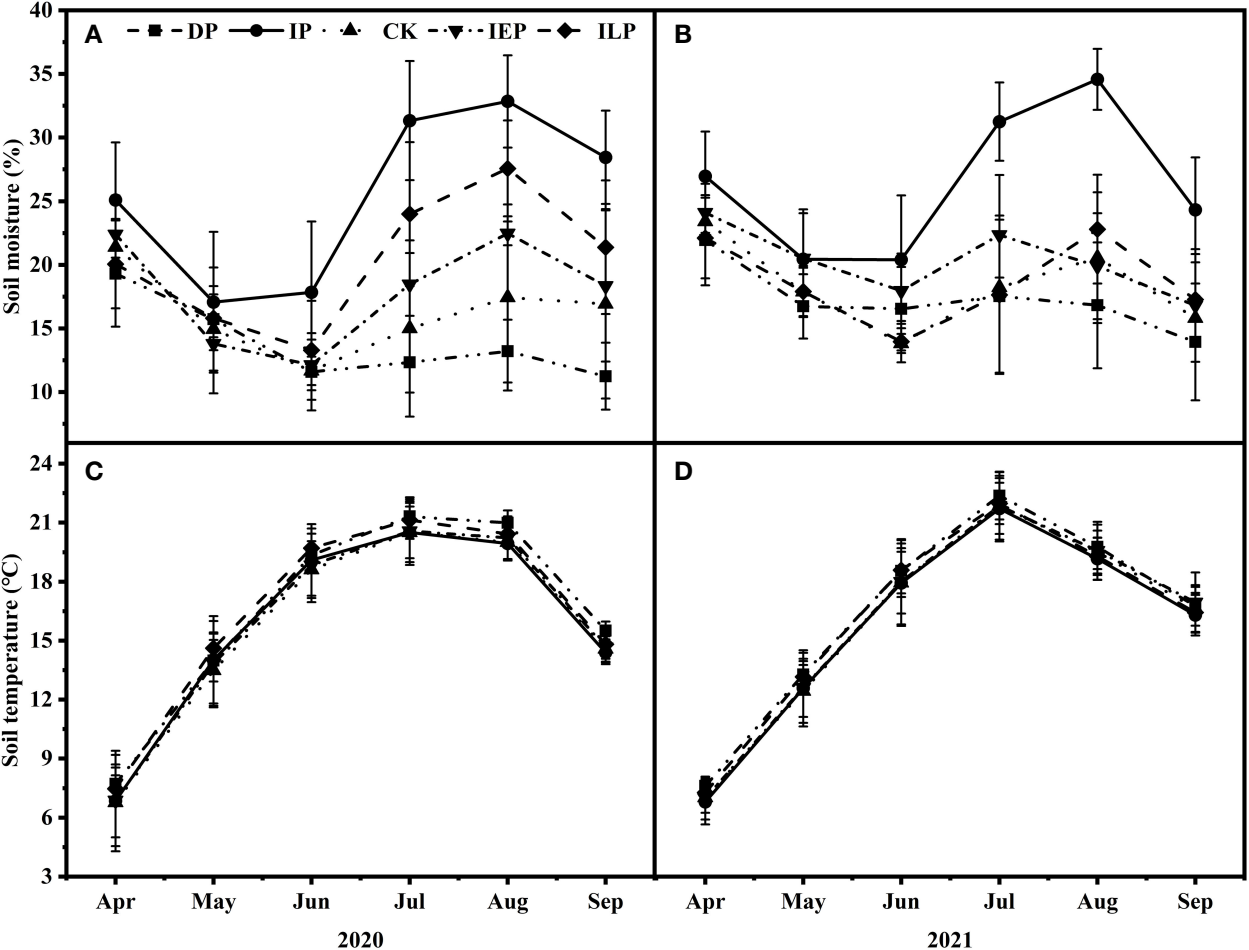
Effects of intra-annual precipitation patterns during the growing seasons (April–September) on **(A, B)** soil moisture and **(C, D)** soil temperature from 2020 to 2021 (means ± SE). Multi-year average precipitation during April–September in control (CK), 50% decrease in precipitation in entire growing season (DP, April–September), 50% increase in precipitation in entire growing season (IP, April–September), increase in the early growing season precipitation by 50% (IEP, April–June), and increase in the late growing season precipitation by 50% (ILP, July–September).

The soil moisture and soil temperature showed significant seasonal variation during the experimental period (**Figure 1**, **Supplementary Table S3**). The snowmelt replenished the soil moisture in all the plots during the early growing season, and precipitation drove the soil moisture differences later in the growing season. Overall, the soil moisture increased by 14.42% and 13.07% under the IP plots in 2020 (**Figure 1A**) and 2021 (**Figure 1B**), respectively, and increased by 7.86% and 3.15% under the ILP plots, respectively. There were no significant differences between the soil temperature for all the experimental plots (**Figures 1C, D**); the maximum temperatures were reached in July and showed consistent variation with the air temperature in this study area.

### Effects of intra-annual precipitation patterns on community phenology and biomass

**Figure 2:**
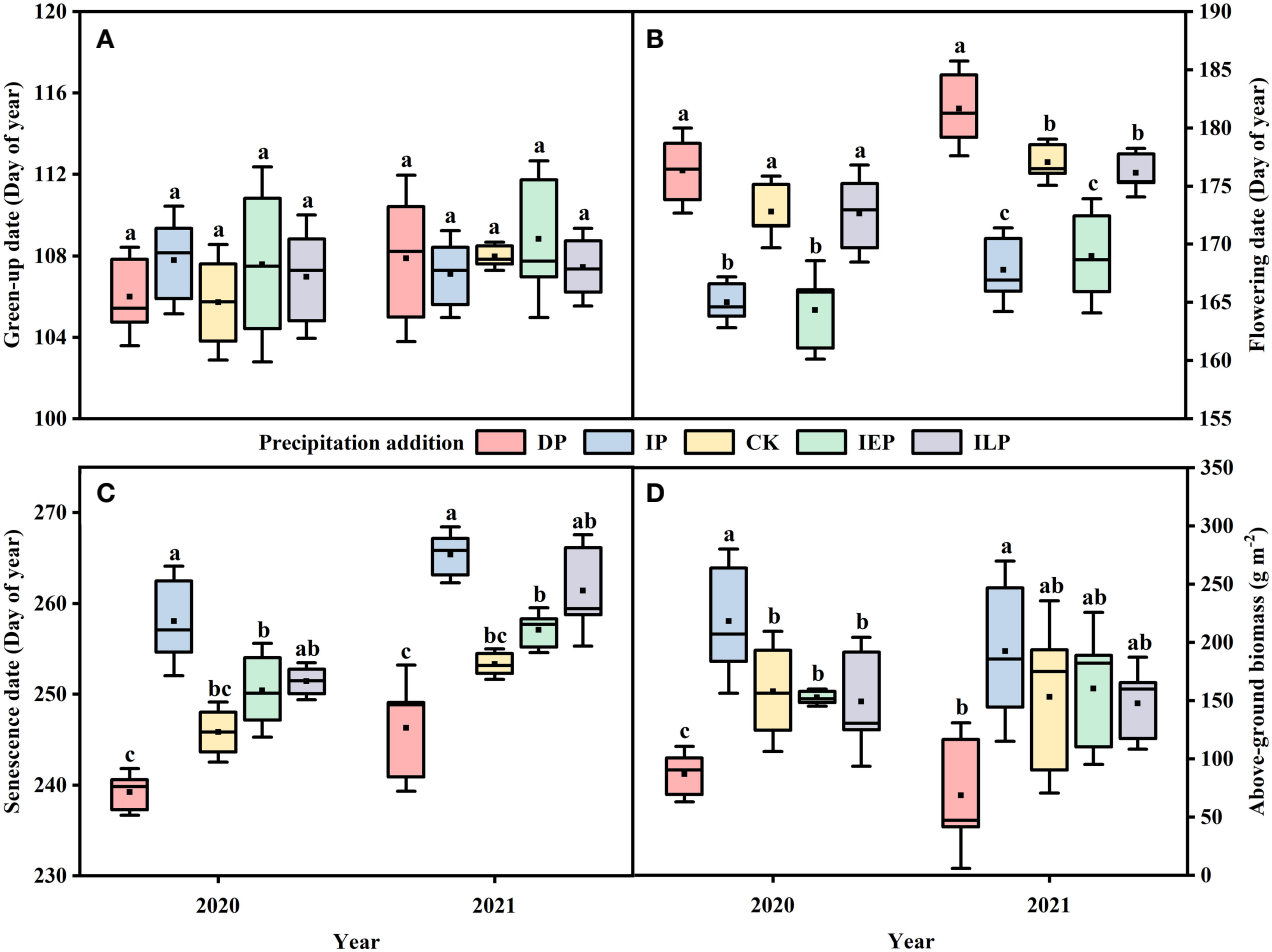
Effects of intra-annual precipitation patterns on community **(A)** green-up, **(B)** flowering, **(C)** senescence, and **(D)** above-ground biomass from 2020 to 2021. The significance of each factor in different treatments was tested by one-way ANOVA. In Tukey’s HSD’s multi-range test, the different letters at the top of each box represent significant differences between different treatments (P < 0.05). Different colors represent the intra-annual precipitation treatment (n = 15, 5 individuals × 3 replicates). The boxes indicate the 25-75% confidence interval of each target variable. The solid lines and black squares inside the box represent the median and mean, respectively. Abbreviations are as in **Figure 1**.

Community green-up was not affected by year, species, intra-annual precipitation patterns, or their interactive effects (**Figure 2A**, **Supplementary Table S4**). However, the intra-annual precipitation patterns, species, year, and the interaction of the species and year had significant effects on community flowering (**Supplementary Table S4**). Compared to the controls plots, the IP plots and IEP plots advanced flowering by 7.8 (± 1.2) and 8.4 (± 2.3) days in 2020 and by 9.3 (± 2.0) and 8.1 (± 2.7) days in 2021 (P < 0.05; **Figure 2B**). In the DP plots, community flowering was delayed by 3.5 (± 2.0) days in 2020 (P > 0.05) and by 4.6 (± 2.2) days in 2021 (P < 0.05; **Figure 2B**). The IP plots’ community senescence was delayed by 12.2 (± 3.3) and 12.1 (± 1.7) days in 2020 and 2021, respectively; in contrast, community senescence in the DP plots was advanced by 6.6 (± 1.4) and 7.1 (± 3.8) days in 2020 and 2021, respectively, compared with the controls plots (P < 0.05; **Figure 2C**).

In addition to these phenological shifts, sustained increases or decreases in precipitation during the growing season had a significant effect on the community above-ground biomass (**Figure 2D**). In the IP plots, the average community above-ground biomass during the experiment was 205.35 (± 33.68) g m^-2^, which was significantly greater than the DP average of 77.71 (± 12.97) g m^-2^ (P < 0.05; **Figure 2D**). Neither the IEP nor the ILP treatment differed significantly from the CK treatment.

### Dominant species phenology and biomass responses to intra-annual precipitation patterns

**Figure 3:**
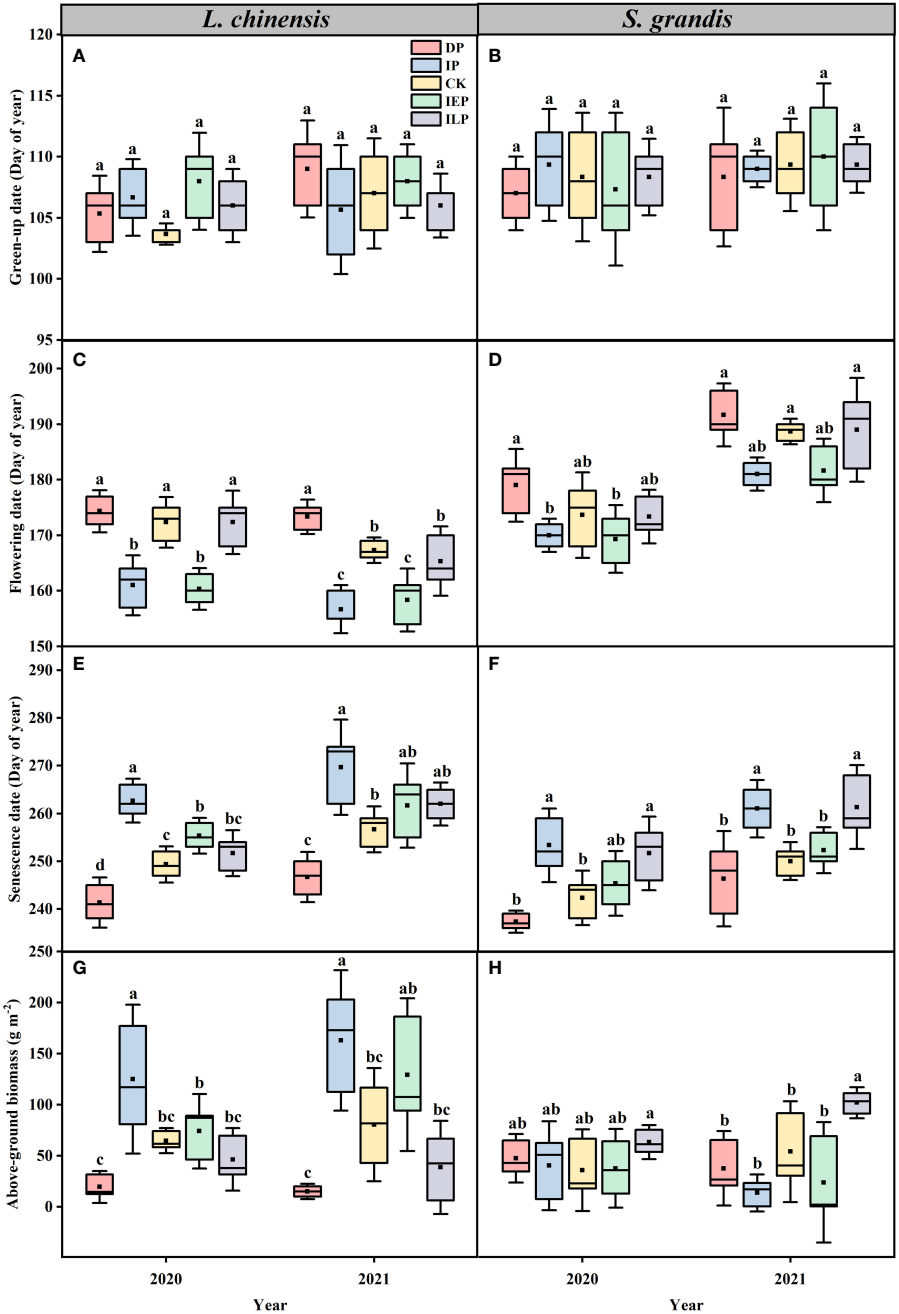
Effects of intra-annual precipitation patterns on the dominant species **(A, B)** green-up, **(C, D)** flowering, **(E, F)** senescence, and **(G, H)** above-ground biomass from 2020 to 2021. The significance of each factor in different treatments was tested by one-way ANOVA. In Tukey’s HSD’s multi-range test, the different letters at the top of each box represent significant differences between the different treatments (P < 0.05). Different colors represent the intra-annual precipitation treatment (n = 15, 5 individuals × 3 replicates). The boxes indicate the 25-75% confidence interval of each target variable. The solid lines and black squares inside the box represent the median and mean, respectively. Abbreviations are as in **Figure 1**.

We found that variations in intra-annual precipitation patterns did not significantly affect the green-up of the dominant species during this experiment (**Supplementary Table S4**); the dominant species, *L. chinensis* and *S. grandis*, showed an average green-up on day 106.5 (± 2.2) and day 108.6 (± 3.1) (**Figures 3A, B**), respectively. However, flowering and senescence for the dominant species varied between the precipitation treatments. Compared to the controls plots, flowering of *L. chinensis* advanced by 11.3 (± 2.9) in 2020 and 10.6 (± 2.4) days in 2021 in the IP plots and by 12 (± 2.1) and 9 (± 3.1) days in the IEP plots in 2020 and 2021, respectively (P < 0.05; **Figure 3C**). Similarly, *S. grandis* showed advanced flowering in the IP and IEP treatments by 7.7 (± 1.6) days and 7 (± 3.2) days in 2021 (P < 0.05), respectively, but there were no significant changes in 2020 (P > 0.05; **Figure 3D**). The average flowering date of *L. chinensis* for all the plots was 13.6 (± 4.2) days ahead of *S. grandis*. Compared with the CK plots, senescence for *L. chinensis* and *S. grandis* was delayed by 13.2 (± 4) and 11 (± 3.7) days, respectively, in the IP plots. The DP plots advanced *L. chinensis* senescence by 8 (± 2.9) and 10 (± 2.8) days in 2020 and 2021, respectively (P < 0.05; **Figure 3E**), but they did not significantly change the timing of senescence for *S. grandis* (**Figure 3F**). For the IEP plots, *L. chinensis* senescence was delayed by 6 (± 2.1) days in 2020 (**Figure 3E**). In contrast, the ILP plots showed delayed *S. grandis* senescence by 9.3 (± 4.2) days and 11.3 (± 4.8) days in 2020 and 2021, respectively (P < 0.05; **Figure 3F**). The average date of senescence of *S. grandis* in all the plots was 5.6 (± 3.7) days ahead of *L. chinensis*.

Additionally, the dominant species showed significant differences in above-ground biomass in response to intra-annual precipitation patterns (**Figures 3G, H**; **Supplementary Table S4**). Compared to the controls plots, the above-ground biomass of *L. chinensis* increased significantly in the IP plots (P > 0.05), while a minor, although significant, increase was also observed in the IEP plots (P < 0.05; **Figure 3G**). The ILP plots increased the above-ground biomass of *S. grandis* in 2021 (P < 0.05; **Figure 3H**). Under variable intra-annual precipitation patterns, there were significant negative correlations between the relative biomass of *L. chinensis* and *S. grandis* and between the relative biomass of perennial rhizome grasses and perennial bunchgrasses during the experiment (P < 0.05; **Supplementary Figure S3**).

### Correlation of environmental factors with phenology and biomass

**Figure 4:**
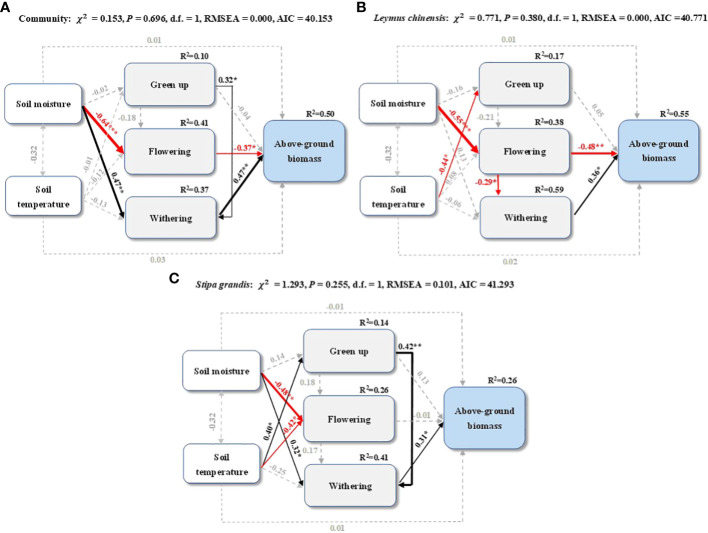
Structural equation models of soil moisture, soil temperature, green-up, flowering, and senescence on the above-ground biomass of **(A)** community, **(B)** *L. chinensis*, and **(C)** *S. grandis*. Black and red arrows represent significant positive and negative pathways, respectively, and grey dashed arrows indicate non-significant pathways. The arrow width is proportional to the strength of the relationship. The numbers adjacent to the arrows are standardized path coefficients and indicate the effect size of the relationship. The proportion of variance explained (R^2^) appears alongside the response variables in the model, and the asterisks indicate statistical significance (*P < 0.05, **P < 0.01, ***P < 0.001).

Using structural equation models, we found that the community and dominant species above-ground biomass responded differently to soil moisture, soil temperature, and phenology (**Figure 4**). Both the community and *L. chinensis* above-ground biomass were negatively affected by flowering and positively by senescence (**Figures 4A, B**). Soil moisture indirectly affected the community above-ground biomass through a negative effect on flowering and a positive effect on senescence (**Figure 4A**). Soil moisture indirectly affected the *L. chinensis* above-ground biomass through a negative effect on flowering (**Figure 4B**). The *S. grandis* above-ground biomass was directly affected by senescence and indirectly by soil moisture (**Figure 4C**). In addition, there were positive effects of green-up on the senescence of the community (**Figure 4A**) and *S. grandis* (**Figure 4C**) and negative effects of flowering on *L. chinensis* senescence (**Figure 4B**).

## Discussion

Intra-annual precipitation variability is a key driver of community biomass (Zhang et al., 2022a), because differences in the timing of precipitation may affect community biomass directly or indirectly through shifts in species composition and phenology. Our results showed that increased precipitation throughout the entire growing season extended the length of the growing season and increased community biomass through advanced community flowering and delayed senescence, while sustained precipitation reduction delayed community flowering and reduced community biomass. In addition, complementary effects (in terms of biomass and phenology) between the dominant species and functional groups maintained the dynamic stability of community biomass under different intra-annual precipitation variations.

Temperature and precipitation are widely recognized as the primary controls for grassland phenology (Forkel et al., 2015; Fu et al., 2021; Post et al., 2022). Plants need to accumulate sufficient growing degree days for green-up and to enhance photosynthetic enzyme activity (Liu et al., 2016; Ganjurjav et al., 2020), and there must be sufficient soil moisture to initiate plant growth (Post et al., 2022). Despite divergent soil moisture patterns between treatments, our results showed no significant differences in the green-up dates at the community level. Previous studies have shown that the green-up of temperate grasslands is mainly influenced by preseason precipitation (Wang et al., 2019; Li et al., 2020; Fu et al., 2021), and since we did not manipulate precipitation during the dormant season, this is likely why we did not see significant differences in green-up dates between treatments at the community level (**Figure 2A**). In fact, soil moisture was generally higher in April for all plots, possibly due to spring snowmelt replenishing the soil water availability (**Figures 1A, B**). Similarly, intra-annual precipitation variation had no significant effects on the green-up dates of either of the dominant species, *L. chinensis* and *S. grandis* (**Figures 3A, B**), which did not significantly differ in the timing of green-up (**Supplementary Table S4**; Li et al., 2016a; Wang et al., 2022). It is worth noting that plant green-up may be a transitory process (Ganjurjav et al., 2020); thus, it is possible that the frequency at which we measured phenology (every seven days) may have contributed to our insignificant differences at the dominant species and community levels.

Community-level response is the integrated product of species-level responses, and community-level phenology is largely dependent on the dominant species (Robertson et al., 2009; Shaw et al., 2022). Our results showed that increased early growing season precipitation advanced the flowering dates of both the community (**Figure 2B**) and the dominant species, *L. chinensis* (**Figure 3C**), while having no effect on *S. grandis* (**Figure 3D**), which is consistent with previous results (Liu et al., 2009; Zhou et al., 2019). It is possible that the differences in flowering dates were the result of competition between the dominant species for soil moisture in the early growing season. Since *S. grandis* roots are the only below-ground organ, their primary function is to acquire water and nutrients. In contrast, *L. chinensis* has rhizomes in addition to roots, which function for vegetative reproduction and storage (Zhao et al., 2008; Liu et al., 2012), as well as greater root biomass, root length, and rhizosphere respiration than *S. grandis* (Lu et al., 2019). Thus, *L. chinensis* could use the limited soil moisture to promote its rhizome development and nutrient expansion, increasing its inorganic and organic resource storage and promoting plant growth (Liu et al., 2009; Fu et al., 2021). Further, *L. chinensis* has a higher rain-use efficiency (RUE) and a more flexible water source than *S. grandis* under water stress, utilizing not only the surface soil moisture but also the deep soil moisture supplemented by winter snowfall, especially in the early growing season (Bai et al., 2008; Yang et al., 2011; Bao et al., 2019). In addition, long-term observational studies have shown that *L. chinensis* requires less thermal time before flowering and has an earlier flowering date compared to *S. grandis* (Li et al., 2016a; Wang et al., 2022). As a result of these differences between the dominant species, an increased early growing precipitation season significantly advanced the *L. chinensis* flowering dates because of enhanced water availability, as corroborated in previous studies (Li et al., 2016b; Fu et al., 2021).

Additionally, autumn phenology plays a critical role in determining the growing season length and controlling energy exchange in grassland ecosystems (Piao et al., 2008; Richardson et al., 2013). Our results showed that increased late growing season precipitation delayed both the community (**Figure 2C**) and the dominant species *S. grandis* (**Figure 3F**) senescence dates, which is consistent with previous studies that found that adequate precipitation could postpone the end of the season (Liu et al., 2016; Ren et al., 2019). In particular, previous work has shown that *S. grandis* mainly absorbs water from the upper soil layer when it is highly available (Yang et al., 2011; Bao et al., 2019), so the additional precipitation during the late season likely delays senescence. Further, *S. grandis* has a higher soil nitrogen uptake rate than *L. chinensis*, and with increased late growing season precipitation, soil ammonium nitrogen content can increase, which could improve *S. grandis* nitrogen-use efficiency (NUE), further promoting its growth and delaying senescence (Gong et al., 2011; Wang et al., 2016). In addition, compared to *L. chinensis*, the water requirement for flowering and the wilting point is higher in *S. grandis* (Wang et al., 2022), which is reflected in the flowering dates. *L. chinensis* often blooms in late June and matures in late July, while *S. grandis* often blooms in early August and matures in mid-August (Zhang et al., 2018). It is worth noting that *L. chinensis* senescence dates were significantly advanced in the plots that had decreased precipitation for the entire growing season (**Figure 3E**), which is consistent with previous *in situ* experiments documenting that water deficit may drive leaf senescence in grasses (Dreesen et al., 2014) by inhibiting plant photosynthesis (Ripley et al., 2007), thus accelerating chlorophyll and protein degradation (Galmés et al., 2007) and constraining stomatal size (Xu and Zhou, 2008).

Phenological dynamics are often then reflected in above-ground productivity, which can be an important indicator for grassland functioning (Knapp et al., 2006; Wilcox et al., 2017). A growing number of studies have shown that intra-annual precipitation patterns are better predictors of productivity response within site than annual precipitation alone, especially for semi-arid ecosystems (Swemmer et al., 2007; Knapp et al., 2008; Hoover et al., 2021; Shaw et al., 2022). Our results showed that at the species level, the early growing season precipitation increased the *L. chinensis* above-ground biomass by advancing its flowering dates (**Figure 3G**, **Figure 4B**), while the late growing season precipitation increased the *S. grandis* above-ground biomass by delaying senescence (**Figure 3H**, **Figure 4C**). Studies have shown that advanced flowering can directly increase the above-ground biomass by extending the plant reproductive length and promoting plant organ production, such as flowering stalks and tiller branches (La Pierre et al., 2011). In addition, advanced flowering could indirectly increase the above-ground biomass by affecting functional traits such as the plant height and leaf area (Gong et al., 2011; Li et al., 2021). Consistent with previous results in this study area, increased late growing season precipitation weakened the effects of water deficit on the plant photosynthetic efficiency, reduced leaf senescence, extended the growing season length, and increased the above-ground biomass (Liu et al., 2016; Ren et al., 2019). Intra-annual precipitation patterns determine the availability of soil moisture at critical plant growth stages, suggesting that soil moisture at specific times may have outsized impacts on the annual above-ground biomass (Knapp et al., 2008; Prevéy and Seastedt, 2014; Zelikova et al., 2015; Wang et al., 2019). Whether these additional water additions increase biomass by causing different dominant species root growth, higher mineralization, or just by an increase in diffusive nitrogen transport through the large amount of water in the soil pore, given the tight coupling of water and nitrogen availability (Fanselow et al., 2011; Gong et al., 2011), is unclear from this study. At the community level, increased precipitation in the early or late growing season did not significantly alter the above-ground biomass compared with the control (**Figure 2D**). However, part of the reason that we did not see differences in the overall above-ground biomass with the early or late growing season water additions was because of the complementary effects between the two dominant species (**Supplementary Figure S3**). Complementarity between the two main dominant species allowed these treatments to maintain a stable community biomass under different intra-annual precipitation patterns (Bai et al., 2004; Hallett et al., 2014; Shaw et al., 2022). Species’ life history strategies determine how intra-annual precipitation patterns affect compensation in terms of the above-ground biomass (Bai et al., 2004), below-ground biomass (Liu et al., 2018), community composition (Hallett et al., 2019), phenology (Cleland et al., 2006), and nitrogen utilization (Gong et al., 2011). Although we saw some evidence of compensation, our precipitation manipulations were short term, and it may take more than four years of precipitation changes in grassland to observe significant changes in the above-ground biomass, as noted in the previous study (Evans et al., 2011).

## Conclusion

Based on a two-year intra-annual precipitation patterns manipulative experiment, we found that increased early growing season precipitation enhanced the above-ground biomass of the dominant rhizome grass, *L. chinensis*, by advancing its flowering date, while increased late growing season precipitation increased the above-ground biomass of the dominant bunchgrass, *S. grandis*, by delaying its senescence. The complementary effects in the phenology and biomass of the dominant species, *L. chinensis* and *S. grandis*, maintained stable dynamics of the community above-ground biomass under intra-annual precipitation pattern variations. We provide evidence that soil moisture plays a critical role in determining temperate grassland phenology responses to intra-annual precipitation patterns. Understanding how plants respond to climate change requires the use of multi-factor, multi-species experiments and long-term observations to better predict ecosystem functions under future climate scenarios.

## Data availability statement

The raw data supporting the conclusions of this article will be made available by the authors, without undue reservation.

## Author contributions

ZZ, YH, TB, and OH raised the scientific questions and designed the experiments. ZHZ, JY, and HQ conducted the experiments. ZZ, ZHZ, and TB analyzed the experimental data. ZZ, OH, and AK wrote the manuscript. All the authors discussed the results and approved the final manuscript and contributed substantially to this research work. All authors contributed to the article and approved the submitted version.
