# Supplementary material for: Effects of intra-annual precipitation patterns on grassland productivity moderated by the dominant species phenology
Source: Front Plant Sci. 2023 Apr 11;14:1142786. doi: 10.3389/fpls.2023.1142786 (PMC10126275; doi:10.3389/fpls.2023.1142786)
Supplement: Supplementary file 1 [file DataSheet_1.docx]

**Supporting information**


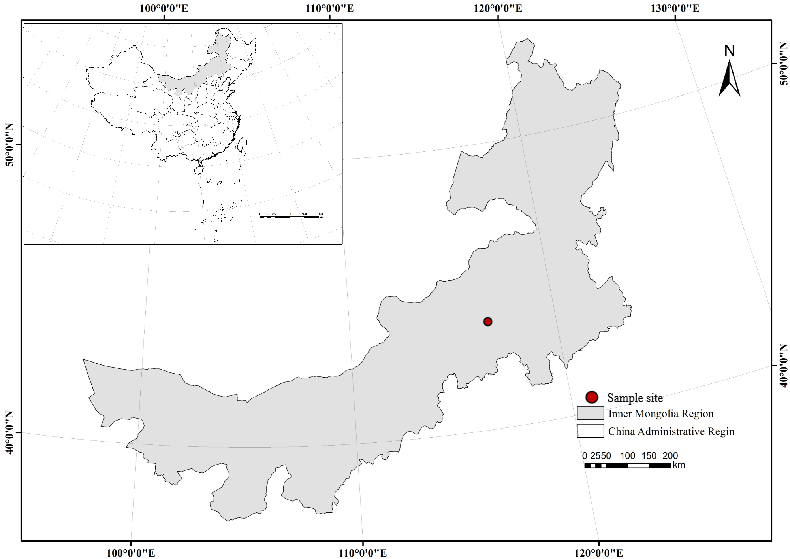


**Figure S1.** Location of sampling area of study region in Inner Mongolia of China.

**Figure S2.** A priori conceptual model for effects of soil moisture and soil temperature on aboveground biomass through shifts in plant phenology including plant green up, flowering and senescence.


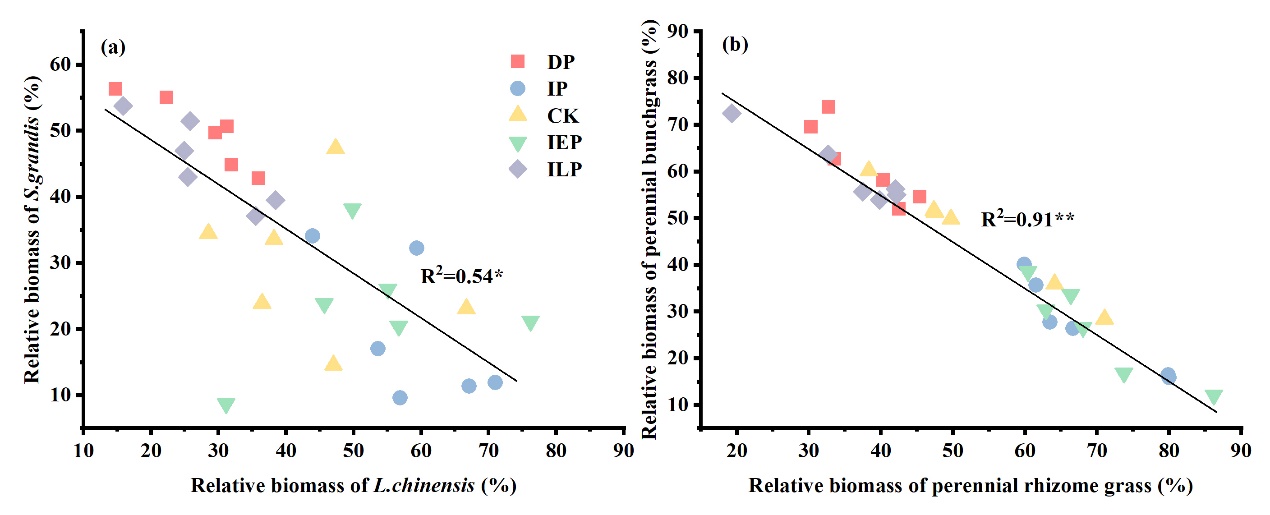


**Figure S3.** Correlation between the relative biomass of (a) dominant species *L. chinensis* and *S. grandis*, (b) functional group perennial rhizome grass and perennial bunchgrass under intra-annual precipitation addition during the experiment. Abbreviations: precipitation during April-September in control (CK), decrease precipitation in entire growing season (DP, April-September), increase precipitation in entire growing season (IP, April-September), increase precipitation in the early growing season (IEP, April-June) and increase precipitation in the late growing season (ILP, July-September). *L. chinensis* means *Leymus chinensis*, *S. grandis* means *Stipa grandis*.

**Table S1.** Common species and their relative abundance within this experimental site.

| Species |  | Functional group |  | Relative abundance |  | Species |  | Functional group |  | Relative abundance |
| --- | --- | --- | --- | --- | --- | --- | --- | --- | --- | --- |
| *Leymus chinensis* |  | PR |  | 41.21 ± 5.3 % |  | *Stipa*  *grandis* |  | PB |  | 35.7 ± 4.8 % |
| *Agropyron cristatum* |  | PR |  | 3.12 ± 0.6 % |  | *Koeleria*  *macrantha* |  | PB |  | 2.52 ± 1.7 % |
| *Carex korshinskii* |  | PR |  | 2.89 ± 0.8 % |  | *Cleistogenes squarrosa* |  | PB |  | 4.51 ± 1.1 % |
| *Klasea centauroides* |  | PF |  | 0.73 ± 0.3 % |  | *Heteropappus altaicus* |  | PF |  | 0.74 ± 0.2 % |
| *Allium anisopodium* |  | PF |  | 0.70 ± 0.03 % |  | *Astragalus galactites* |  | PF |  | 0.44 ± 0.04 % |
| *Thalictrum petaloideum* |  | PF |  | 0.47 ± 0.04 % |  | *Allium tenuissimum* |  | PF |  | 1.29 ± 0.07 % |
| *Medicago ruthenica* |  | PF |  | 0.31 ± 0.03 % |  | *Potentilla tanacetifolia* |  | PF |  | 0.28 ± 0.02 % |
| *Artemisia frigida* |  | SS |  | 2.31 ± 0.1 % |  | *Salsola*  *collina* |  | AB |  | 0.7 ± 0.3 % |
| *Dysphania aristata* |  | AB |  | 0.38 ± 0.01 % |  | *Axyris amaranthoides* |  | AB |  | 0.35 ± 0.1 % |
| *Chenopodium album* |  | AB |  | 0.67 ± 0.2 % |  | *Dontostemon micranthus* |  | AB |  | 0.68 ± 0.2 % |

Abbreviations: perennial rhizome grass (PR), perennial bunchgrasses (PB), perennial forbs (PF), shrubs and semi-shrubs (SS), and annuals and biennials (AB).

**Table S2.** Date and amount (mm) of irrigation for each treatment during the experiment.

| Date |  | Treatment | | | | |  | Date |  | Treatment | | | | |
| --- | --- | --- | --- | --- | --- | --- | --- | --- | --- | --- | --- | --- | --- | --- |
|  |  | CK | DP | IP | IEP | ILP |  |  |  | CK | DP | IP | IEP | ILP |
| 4-7 |  | 1.82 | 0.91 | 2.73 | 2.73 | 1.82 |  | 4-9 |  | 0.38 | 0.19 | 0.57 | 0.57 | 0.38 |
| 4-18 |  | 1.13 | 0.57 | 1.70 | 1.70 | 1.13 |  | 4-21 |  | 0.78 | 0.39 | 1.17 | 1.17 | 0.78 |
| 5-5 |  | 9.46 | 4.73 | 14.19 | 14.19 | 9.46 |  | 5-10 |  | 2.42 | 1.21 | 3.63 | 3.63 | 2.42 |
| 5-20 |  | 4.87 | 2.44 | 7.31 | 7.31 | 4.87 |  | 5-26 |  | 4.44 | 2.22 | 6.66 | 6.66 | 4.44 |
| 5-28 |  | 2.11 | 1.06 | 3.17 | 3.17 | 2.11 |  | 5.31 |  | 2.84 | 1.42 | 4.26 | 4.26 | 2.84 |
| 6-10 |  | 6.81 | 3.41 | 10.22 | 10.22 | 6.81 |  | 6-14 |  | 7.04 | 3.52 | 10.56 | 10.56 | 7.04 |
| 6-15 |  | 1.63 | 0.82 | 2.45 | 2.45 | 1.63 |  | 6-16 |  | 1.02 | 0.51 | 1.53 | 1.53 | 1.02 |
| 6-20 |  | 8.47 | 4.24 | 12.71 | 12.71 | 8.47 |  | 6-21 |  | 2.27 | 1.14 | 3.41 | 3.41 | 2.27 |
| 6-23 |  | 5.96 | 2.98 | 8.94 | 8.94 | 5.96 |  | 6-28 |  | 8.98 | 4.49 | 13.47 | 13.47 | 8.98 |
| 6-29 |  | 2.24 | 1.12 | 3.36 | 3.36 | 2.24 |  | 6-30 |  | 1.63 | 0.82 | 2.45 | 2.45 | 1.63 |
| 7-8 |  | 17.49 | 8.75 | 26.24 | 17.49 | 26.24 |  | 7-10 |  | 4.33 | 2.17 | 6.50 | 4.33 | 6.50 |
| 7-12 |  | 3.15 | 1.58 | 4.73 | 3.15 | 4.73 |  | 7-14 |  | 8.34 | 4.17 | 12.51 | 8.34 | 12.51 |
| 7-15 |  | 2.03 | 1.02 | 3.05 | 2.03 | 3.05 |  | 7-18 |  | 8.43 | 4.22 | 12.65 | 8.43 | 12.65 |
| 7-19 |  | 3.31 | 1.66 | 4.97 | 3.31 | 4.97 |  | 7-23 |  | 4.80 | 2.40 | 7.20 | 4.80 | 7.20 |
| 7-25 |  | 4.51 | 2.26 | 6.77 | 4.51 | 6.77 |  | 7-26 |  | 2.02 | 1.01 | 3.03 | 1.01 | 3.03 |
| 7-28 |  | 4.88 | 2.44 | 7.32 | 4.88 | 7.32 |  | 7-31 |  | 7.18 | 3.59 | 10.77 | 7.18 | 10.77 |
| 8-5 |  | 9.47 | 4.74 | 14.21 | 9.47 | 14.21 |  | 8-9 |  | 6.39 | 3.20 | 9.59 | 6.39 | 9.59 |
| 8-10 |  | 2.45 | 1.23 | 3.68 | 2.45 | 3.68 |  | 8-11 |  | 6.37 | 3.19 | 9.56 | 6.37 | 9.56 |
| 8-17 |  | 12.11 | 6.06 | 18.17 | 12.11 | 18.17 |  | 8-18 |  | 2.40 | 1.20 | 3.60 | 2.40 | 3.60 |
| 8-19 |  | 1.33 | 0.67 | 2.00 | 1.33 | 2.00 |  | 8.24 |  | 7.11 | 3.56 | 10.67 | 7.11 | 10.67 |
| 8-27 |  | 6.55 | 3.28 | 9.83 | 6.55 | 9.83 |  | 8-30 |  | 4.05 | 2.03 | 6.08 | 4.05 | 6.08 |
| 9-1 |  | 1.97 | 0.99 | 2.96 | 1.97 | 2.96 |  | 9-3 |  | 2.22 | 1.11 | 3.33 | 2.22 | 3.33 |
| 9-7 |  | 4.60 | 2.30 | 6.90 | 4.60 | 6.90 |  | 9-9 |  | 4.67 | 2.34 | 7.01 | 4.67 | 7.01 |
| 9-16 |  | 4.04 | 2.02 | 6.06 | 4.04 | 6.06 |  | 9-24 |  | 4.13 | 2.07 | 6.20 | 4.13 | 6.20 |
| **Total** |  | **233.28** | **116.64** | **349.92** | **271.43** | **311.77** |  |  |  |  |  |  |  |  |

Abbreviations: precipitation during April-September in control (CK), decrease precipitation in entire growing season (DP, April-September), increase precipitation in entire growing season (IP, April-September), increase precipitation in the early growing season (IEP, April-June) and increase precipitation in the late growing season (ILP, July-September).

**Table S3.** Result of repeated measured analysis of variance (RMANOVA) for main and interactive effects of year (Y), growth stage (G), and intra-annual precipitation addition (P) on soil moisture and soil temperature. The *p* values lower than 0.05 are bolded.

| Factors |  | Soil moisture | |  | Soil temperature | |  |
| --- | --- | --- | --- | --- | --- | --- | --- |
|  |  | *F* | *P* |  | *F* | *P* |  |
| P |  | 38.21 | ˂ **.001** |  | 11.09 | .058 |  |
| G |  | 9.03 | ˂ **.001** |  | 79.32 | ˂ **.001** |  |
| Y |  | 0.32 | .590 |  | 5.71 | .075 |  |
| P × G |  | 3.04 | **.002** |  | 0.67 | .702 |  |
| P × Y |  | 2.61 | .072 |  | 1.04 | .359 |  |
| G × Y |  | 0.53 | .781 |  | 1.53 | .218 |  |
| P × G × Y |  | 0.89 | .602 |  | 0.28 | .964 |  |

**Table S4.** Result of repeated measured analysis of variance (RMANOVA) for main and interactive effects of year (Y), species (S), and intra-annual precipitation addition (P) on community green-up, flowering, senescence, and biomass. The *p* values lower than 0.05 are bolded.

| Factors |  | Green-up  date | |  | Flowering  date | |  | Senecsence  date | |  | Above-ground biomass | |
| --- | --- | --- | --- | --- | --- | --- | --- | --- | --- | --- | --- | --- |
|  |  | *F* | *P* |  | *F* | *P* |  | *F* | *P* |  | *F* | *P* |
| P |  | 0.54 | 0.707 |  | 28.84 | **˂ 0.001** |  | 36.15 | **˂ 0.001** |  | 6.11 | **0.001** |
| S |  | 4.07 | 0.078 |  | 229.6 | **˂ 0.001** |  | 39.91 | **˂ 0.001** |  | 11.27 | **0.010** |
| Y |  | 1.26 | 0.295 |  | 27.81 | **0.001** |  | 85.46 | **˂ 0.001** |  | 1.86 | 0.210 |
| P × S |  | 1.22 | 0.320 |  | 1.65 | 0.186 |  | 2.92 | **0.036** |  | 15.32 | **˂ 0.001** |
| P × Y |  | 1.02 | 0.411 |  | 0.22 | 0.927 |  | 0.33 | 0.859 |  | 0.84 | 0.511 |
| S × Y |  | 0.5 | 0.781 |  | 91.81 | **˂ 0.001** |  | 0.48 | 0.508 |  | 0.88 | 0.376 |
| P × S × Y |  | 0.75 | 0.565 |  | 0.94 | 0.454 |  | 0.09 | 0.984 |  | 3.19 | **0.026** |
